# Exopolysaccharides Produced by Lactic Acid Bacteria: From Biosynthesis to Health-Promoting Properties

**DOI:** 10.3390/foods11020156

**Published:** 2022-01-08

**Authors:** Dominika Jurášková, Susana C. Ribeiro, Celia C. G. Silva

**Affiliations:** Institute of Agricultural and Environmental Research and Technology (IITAA), University of the Azores, 9700-042 Angra do Heroísmo, Azores, Portugal; dominika.juraskova@gmail.com (D.J.); susana.ic.ribeiro@uac.pt (S.C.R.)

**Keywords:** exopolysaccharides, EPS, lactic acid bacteria, LAB, structure, biosynthesis, food application, health, prebiotics, probiotics

## Abstract

The production of exopolysaccharides (EPS) by lactic acid bacteria (LAB) has attracted particular interest in the food industry. EPS can be considered as natural biothickeners as they are produced in situ by LAB and improve the rheological properties of fermented foods. Moreover, much research has been conducted on the beneficial effects of EPS produced by LAB on modulating the gut microbiome and promoting health. The EPS, which varies widely in composition and structure, may have diverse health effects, such as glycemic control, calcium and magnesium absorption, cholesterol-lowering, anticarcinogenic, immunomodulatory, and antioxidant effects. In this article, the latest advances on structure, biosynthesis, and physicochemical properties of LAB-derived EPS are described in detail. This is followed by a summary of up-to-date methods used to detect, characterize and elucidate the structure of EPS produced by LAB. In addition, current strategies on the use of LAB-produced EPS in food products have been discussed, focusing on beneficial applications in dairy products, gluten-free bakery products, and low-fat meat products, as they positively influence the consistency, stability, and quality of the final product. Highlighting is also placed on reports of health-promoting effects, with particular emphasis on prebiotic, immunomodulatory, antioxidant, cholesterol-lowering, anti-biofilm, antimicrobial, anticancer, and drug-delivery activities.

## 1. Introduction

It is well-known that lactic acid bacteria (LAB) can synthesize a variety of polysaccharides. These comprise a large group of high-molecular-weight molecules consisting of monosaccharide units linked by a glycosidic bond, which exhibit a variety of structures, functional properties, and biological activities [1,2]. Polysaccharides are one of the main components involved in the formation of the extracellular biofilm matrix. They play an important role not only in protecting bacteria from adverse environmental factors, but also in the attachment of microbial cells to solid surfaces [3,4,5,6]. The polysaccharides produced by bacteria can be found in structures on the cell surface classified as capsular polysaccharides or lipopolysaccharides [7]. They form the outermost layer of the bacterial cell and provide a mechanism to protect the cell, mediating direct interactions with the environment. Due to their attachment to the cell surface, they are unlikely to be isolated or separated from the cell biomass, although some capsular polysaccharides can be released from the cell. In contrast, exopolysaccharides are polysaccharides that are loosely associated with the cell surface or released into the extracellular medium [8,9]. They can either be produced extracellularly by enzymes secreted by the bacterium, or synthesized intracellularly and secreted outside the cells [10]. Considering the protective mechanisms for the microbial cell, such as protection against abiotic or biotic stress, competition, pH, and temperature, exopolysaccharides (EPS) have particular physicochemical properties that most likely have potential for the food and pharmaceutical industries. Nevertheless, most of the research is focused on the application of EPS in the food industry due to their structural properties, such as emulsification, texturization, sweetening, gelling, water-binding capacity, or bioactive properties. This is one of the reasons why EPS has attracted the attention of researchers. Recent studies have also demonstrated the health-promoting potential of EPS, including immunomodulatory, prebiotic, anti-inflammatory, anti-biofilm, and antioxidant activities [11,12,13,14,15]. In this review, the structure of EPS produced by LAB, their biosynthesis, and the methods currently used to isolate and characterize EPS are described. In addition, some food and pharmaceutical applications of EPS are discussed, as well as the recent evidence of health-promoting effects of EPS.

## 2. Structure of Exopolysaccharides (EPS)

Bacterial EPSs have diverse and complex chemical structures that strongly influence their functional properties and biological functions. They can be divided into homopolysaccharides (HoPS), which contain a single type of monosaccharide, and heteropolysaccharides (HePS), which consist of two or more types of monosaccharides [16,17,18]. Most of the EPS produced by LAB are HePS and are synthesized intracellularly, while some LAB species/strains produce HoPS by extracellular enzymes [10,19]. The HoPS produced by LAB (Table 1) can be classified as glucans, fructans, or galactans, which consist of D-glucose, D-fructose, or D-galactose, respectively [10,20]. Glucans consist of glucose residues as the main backbone structure with different degrees of branching and binding sites that vary from bacterial strain to bacterial strain. Glucans can be classified as either α-glucans or β-glucans, and are produced by a variety of LAB species in the genera *Leuconostoc*, *Lactobacillus*, *Streptococcus,* and *Weissella* [21]. Alpha-glucans can be divided into four groups: (i) dextran is water-soluble and mostly has α-(l→6) bonds, although some branching may occur at α-(l→2), α-(l→3) or α-(l→4) [22,23]; (ii) mutan is generally water-insoluble and contains mainly α-(l→3) bonds with branching at α-(l→6) [21]; (iii) reuteran is a water-soluble branched α-glucan consisting of α-(l→4)-linear fragments linked by α-(l→6) bonds [24]; and (iv) alternan exhibits alternating α-(l→3) and α-(l→6) bonds and shows lower viscosity and higher solubility in water [25]. Beta-glucans are also HoPS, produced by *Pediococcus* and *Streptococcus* spp., and consist of D-glucose linked by β-(l→3) bonds together with β-(l→2) branches [19,26]. Fructans are water-soluble fructose polymers produced by strains of *Streptococcus salivarius*, *Leuconostoc mesenteroides*, *Limosilactobacillus reuteri* (formerly *Lactobacillus reuteri*), *Lactobacillus johnsonii* and *Fructilactobacillus sanfranciscensis* (formerly *Lactobacillus sanfranciscensis*). They can be divided into (i) levan with β-2,6 link(ages and (ii) inulin with β-2,1 linkages [27]. The water-soluble galactans are less abundant, have α-(1→6)-linked galactose units, and are produced by a few LAB strains belonging to *Weissella confusa*, *Lactococcus lactis* subsp. *lactis* and *Lactobacillus delbrueckii* subsp. *bulgaricus* [19,28].

HePS (Table 2), unlike HoPS, have a more complex structure as they are composed of several repeating units of sugars, such as pentose (D-ribose, D-arabinose, D-xylose), hexose (D-glucose, D-galactose, D-mannose), N-acetylated monosaccharides (N-acetyl-glucosamine and N-acetyl-galactosamine), or uronic acids (D-glucuronic acid, D-galacturonic acid), and may be branched or unbranched [8,60,61]. They are produced by members of the genera *Lactobacillus*, *Lactococcus*, and *Streptococcus* [10]. HePS are produced in relatively small amounts by LAB but exhibit high thickening power at low concentrations [20,62,63]. Kefiran is an example of HePS produced by several *Lactobacillus* species in kefir grains, including *L. kefiranofaciens*, *L. kefirgranum*, *L. parakefir*, *L. kefir*, and *L. delbrueckii* subsp. *bulgaricus* [64]. Kefiran is a water-soluble branched glucogalactan with a complex structure consisting of D-glucose (Glc) and D-galactose (Gal) in approximately equal amounts, with (1→6)-linked Glc, (1→3)-linked Gal, (1→4)-linked Gal, (1→4)-linked Glc, and (1→2, 6)-linked Gal [65]. Because of these types of linkages, kefiran cannot be hydrolyzed by the digestive enzymes of the human gastrointestinal tract, but it can be fermented by colon bacteria [64]. Other water-soluble HePS include gellan and xanthan, but they are produced by non-LAB, such as *Sphingomonas paucimobilis* and *Xanthomonas campestris* [66].

## 3. EPS Biosynthesis

Regardless of their different structures, the biosynthetic pathways of EPS are remarkably similar between different species of microorganisms [88,89,90]. It is known that HoPS are synthesized by only one enzyme encoded by one gene. In contrast, the genetic sequence of HePS encodes multiple glycosyltransferases, polymerization proteins, and regulatory proteins [90,91]. From a biochemical point of view, there are two major groups of enzymes involved in the production of EPS. The first group consists of enzymes necessary for the synthesis of basic sugar nucleotides used by other cellular metabolic pathways [92,93]. The second group consists of EPS-specific enzymes, such as glycosyl- and acetyltransferases or enzymes responsible for polymerization and export, that regulate the whole process. However, there are also some EPS-specific enzymes whose function is not yet known [92,94]. EPS-specific enzymes are regulated by genes that are usually arranged in clusters on chromosomes or plasmids [55,95,96]. The biosynthesis of HoPS is a relatively simple process because there are no active transport steps and no unnecessary energy is consumed except for the biosynthesis of the extracellular enzymes. Therefore, HoPS is usually synthesized extracellularly mainly from sucrose, although recent reports describe the use of maltodextrins and starch substrates as donors of glucosyl units by some LAB species/strains [8,21]. The enzyme that catalyzes the polymerization of HoPS is a glycosylhydrolase that cleaves the glycosidic bond of its substrate (sucrose) and couples the glucosyl or fructosyl units to synthesize either α-glucans or β-fructans. The energy released by cleavage of the energetic glycosidic bond is used to transfer the monosaccharide units to the reducing end of the polymer [8,97,98,99,100]. However, β-glucans are synthesized intracellularly by a membrane-associated glucosyltransferase [19]. The process of biosynthesis of HePS is more extensive and energy-consuming. The reaction steps for the synthesis of HePS are as follows: (I) internalization of the sugar, (II) synthesis of sugar nucleotide precursors from glucose-1-phosphate and fructose-6-phosphate, which provide the energy for the polymerization reaction, (III) transport across the membrane via a flippase, and (IV) polymerization by various types of glycosyltransferases located at the cytoplasmic membrane adds the monosaccharide moiety to the reducing end of the chain [90,92,101,102]. Due to variability in composition and molecular weight, HePS exhibits large differences in their stiffness, charges, spatial arrangement, and ability to interact with proteins. The variability in composition and molecular weight also affects the physicochemical properties, such as viscosity and solubility [8,36,103,104]. As for the LAB production of EPS, the yield is generally higher for HoPS. Several factors can affect the yield of exopolysaccharides, such as the initial pH, temperature, incubation time, composition of the medium, and so forth [105]. Numerous studies indicate that optimization of various conditions during the production of exopolysaccharides can lead to an increase in exopolysaccharide yield [106,107]. The production of exopolysaccharides by lactic acid bacterial strains varies from 10 mg/L to 400 mg/L if the production process is not optimized. In the case of optimization, this amount can multiply [60,108].

## 4. Methods of EPS Screening, Isolation and Characterization

**Figure 1:**
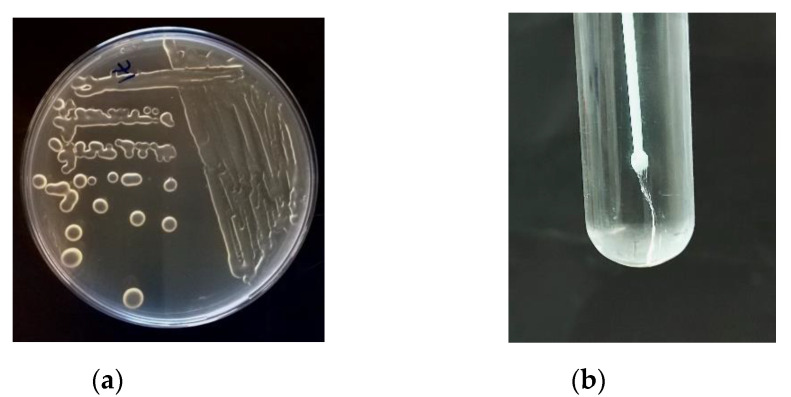
Characteristics of EPS produced by LAB: (**a**) Mucoid colonies—slimy phenotype; (**b**) Ropy phenotype.

Despite the large number of researchers working on EPS-producing LAB, there is still insufficient information on the kinetics of EPS synthesis, where variations in production yield and EPS composition can lead to complete loss of production, as numerous variables can affect production and structure [95,109]. These variables include cultivation time, environmental factors, and carbon source, as they can affect the chemical composition, structure, and even biological properties of EPS [110]. In addition, each microorganism has its own requirements for its ability to produce EPS [2,6,55,111]. Applying the correct methodology for EPS screening, isolation, and purification is critical because, as mentioned previously, EPS production is strain-specific and often depends on the composition of the surrounding media and incubation conditions (e.g., temperature, humidity). A variety of methods for studying EPS produced by LAB, including screening, extraction, and assessment of composition and structure, have been developed and optimized in recent years. The screening methods to evaluate the microbial ability to produce EPS are based on the cultivation of the LAB in a medium enriched with different sugars (glucose, fructose, sucrose, galactose, or lactose). The easiest way to assess EPS production is to visually observe the phenotypic characteristics of the colonies: slimy or ropy phenotypes. The slimy phenotype is characterized by mucilaginous colonies, while the ropy phenotype is characterized by the formation of long filaments when an inoculation loop is lifted from the colony surface or cell pellet (Figure 1). For a more objective test, ruthenium red staining is used in milk agar plates and produces red colonies of non-ropy EPS producers, while ropy colonies remain white [112].

Qualitative methods, such as confocal laser scanning microscopy (CLSM) and electron microscopy (EM), are usually used for the detection of EPS in food. CLSM is used to study the presence and distribution of fluorescently labeled EPS in food matrices and has the advantage of visualizing changes in food microstructures without the need for sample preparation [74]. In contrast, EM requires high vacuum conditions and dehydration of the sample. However, EM is useful to determine the structural features at nanometer scale due to the high resolution of the images produced [74].

**Figure 2:**
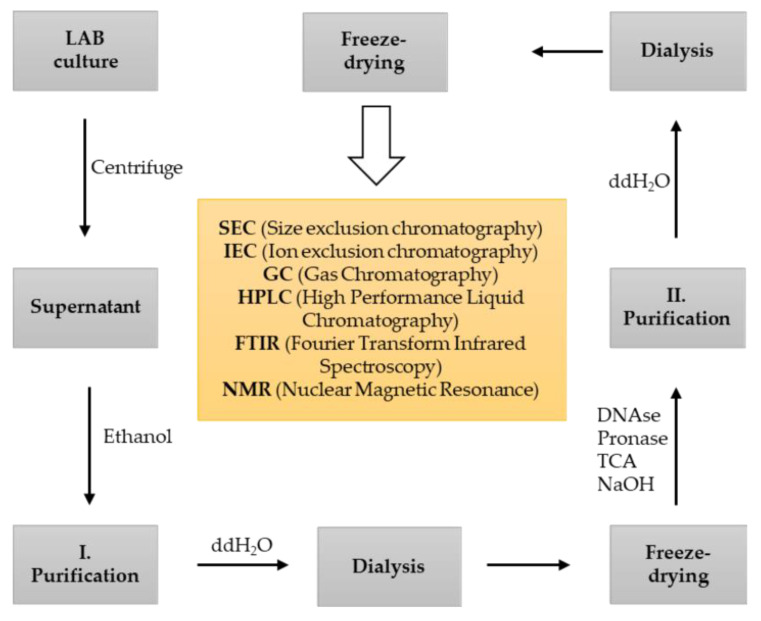
Flow chart for isolation, purification steps (I—first purification, II—second purification), and characterization of EPS.

Quantitative evaluation of EPS produced by LAB requires extraction from the culture media or food matrices. Extraction of EPS involves several steps, including centrifugation to recover the EPS-containing supernatant, addition of acid (e.g., TCA) to remove the high protein content, and precipitation with cold ethanol (Figure 2). The precipitated EPS is then collected and dissolved in water before membrane filtration (usually dialysis or ultrafiltration) to remove the small neutral sugars, salts, and small proteins [19,113,114,115,116]. Finally, the dialyzed EPS solution is freeze-dried to obtain a pure EPS solid, which has a white color and a soft, spongy texture (Figure 2). In this step, the weight of the obtained EPS gives an approximate indication of the EPS yield [117]. Colorimetric methods such as the phenol-sulfuric acid method [118] or the sulfuric acid-UV method, which is more accurate and faster, are usually used to quantify the EPS [119]. Sometimes a second purification step is required to ensure the high purity of EPS [110]. This may involve treatment with proteases and nucleases to remove contaminating proteins and DNA. In addition, anion exchange chromatography and/or size exclusion chromatography are recommended as they contribute to high-purity samples [120]. The number of steps used for EPS isolation depends on the complexity of the media used for production and the type of EPS (HoPS or HePS). Therefore, a prior study is essential to optimize and improve the fermentation conditions.

A complete description of the structural features of EPS includes the identification of monosaccharide composition, ring conformation, linkage, degree of branching, and molecular weight. Several methods have been used to identify and analyze EPS molecular weight, composition, and structure. No single method is capable of assigning all of these parameters, so a combination of several techniques is usually required. Among the most commonly used methods for the determination of EPS molecular mass are size exclusion chromatography (SEC) and ion exclusion chromatography (IEC) [74]. To evaluate the monomer composition, the glycosidic bonds must be previously hydrolyzed by acids or enzymes, and the released monomers are then subjected to high-performance liquid chromatography (HPLC) with refractive index detection (HPLC-RI), high-performance ion exchange chromatography (HPAEC) with pulse amperometric detection (HPAEC-PAD), or gas chromatography [1] coupled with mass spectrometry (GC-MS) [121]. The properties and functionality of EPS also depend on the functional groups and the nature of the bond, which can be detected by Fourier transform infrared spectroscopy (FTIR). Nuclear magnetic resonance (NMR) spectroscopy is also used to analyze the glycosidic bonds, ring configuration, and anomeric configuration of the monomeric units [74,92,122].

## 5. Application of EPS-Producing LAB in Food Products

### 5.1. Yoghurt

EPS produced by LAB can be used to thicken and stabilize fermented dairy products. Lowering the pH during the fermentation process of yoghourt can lead to syneresis resulting from destabilisation of casein micelles. In situ production of EPS has been used to overcome this problem, as it leads to better rheological properties than when EPS is added as one of the components [92,123]. The starter cultures used for yoghourt production, *Lactobacillus delbruecki* subsp. *bulgaricus* and *Streptococcus thermophilus*, were selected to produce exopolysaccharides, generally 60–150 mg/L and 30–890 mg/L, respectively [8,124]. In recent years, studies have been conducted on new EPS-producing starter or adjunct cultures to be used in yoghourt production [8,125]. For example, Han et al. [126] evaluated 19 high EPS-producing *Str. thermophilus* strains isolated from traditional Chinese fermented milk products and used in yoghourt production. They selected a starter with high EPS production (SH-1), which exhibited lower syneresis, better texture, and better sensory evaluation than the samples fermented with a commercial yoghourt starter culture [126]. Fermented dairy products with low fat content can also be improved by using starters with high EPS production, as milk fat plays an important role on the taste, texture, and overall rheological properties of these products [127]. For example, the performance of EPS-producing *Limosilactobacillus mucosae* (formerly *Lactobacillus mucosae*) DPC 6426 as an adjunct culture in the production of low-fat yoghourt has been evaluated and shown to reduce syneresis and improve the functional properties of yoghourt [128].

### 5.2. Cheese

A variety of LAB cultures are used in cheese-making as starters and/or adjunct cultures [129]. Nowadays, customers are looking for healthier, low-fat cheeses, and this is where bacterial strains that can produce EPS come into play. As with any other dairy product, the amount of fat is critical to the texture and flavor of the cheese. Therefore, EPS produced by starter/adjuvant cultures can be used as a fat substitute and texturizer in the production of low-fat cheese [8]. Several studies have shown that EPS improves the texture and quality of low-fat cheese, resulting in a product that has similar properties to its full-fat counterpart. For example, EPS-producing strains of the genus *Lactobacillus* have been shown to increase moisture content and improve the melting of low-fat mozzarella cheese [8,130]. In a study by Costa et al. [131], an EPS-producing strain of *Lactococcus lactis* was used on semi-fat cheddar cheese. Several positive effects of the exopolysaccharide were observed, such as an increase in cheese yield. There was no negative interaction between the starter strain or the ripening strain and the exopolysaccharide-producing strain. After 3 months of ripening, some tests showed that the addition of the EPS-producing strain resulted in a semi-fat cheddar cheese with similar characteristics to a full-fat cheddar cheese without any change in taste [131]. For low-fat cheddar cheese, the addition of the EPS-producing culture of *Lb. plantarum* resulted in cheeses with higher moisture content, higher proteolysis and better sensory values, as well as lower hardness and cohesion compared to the control cheese [132]. In addition, the use of a mixed starter culture containing EPS-producing strains in the production of low-fat Kasar cheese improved the textural properties by producing a less compact protein matrix and a spongy structure [133]. The type of EPS was also shown to affect the rheological properties of the cheese. It was shown that the lower branching ropy EPS reduced protein particle size and decreased creaminess in a model of a low-fat fresh cheese, while the capsular EPS mainly contributed to the reduction of syneresis [134]. The use of EPS-producing LAB in full-fat cheeses has also been studied by several authors. In the production of Prato cheese, the use of exopolysaccharide-producing cultures was shown to improve yield and increase moisture content without affecting proteolysis, pH, melting ability, and sensory acceptability [135]. Rehman et al. [136] used a high EPS-producing strain of *Lactobacillus kefiranofaciens* isolated from Tibetan kefir grain to improve the chewiness and hardness of mozzarella cheese. In a recent study, in situ production of EPS was used to improve the production yield and rheological properties (hardness, elasticity and adhesiveness) of sour whey cheese-requesón [137].

### 5.3. Kefir

Kefir is a self-carbonated, slightly alcoholic beverage made from fermented milk, traditionally from Eastern Europe. Kefir preparation requires kefir grains, which are composed of proteins and polysaccharides and contain a symbiotic association of homofermentative and heterofermentative lactic acid bacteria, acetic acid bacteria, and yeasts [127]. During the fermentation process, exopolysaccharides known as kefiran are produced and act as viscosity regulators. Kefiran is a branched water-soluble glucogalactan composed of equal parts glucose and galactose, and its production is mainly due *to Lactobacillus kefiranofaciens* [8,127]. Within the complex community of kefir grains, other species of LAB have been isolated from kefir and found to produce EPS, such as *Lactiplantibacillus plantarum*, *Lacticaseibacillus paracasei*, *Lactobacillus helveticus*, *Lactiplantibacillus pentosus*, *Lactococcus lactis* subsp. *Lactis*, and *Leuconostoc mesenteroides* [138,139,140,141]. In the last decade, selected strains of mesophilic LAB have been investigated for their ability to ferment milk and produce EPS to improve the rheological properties of fermented milk-based foods [83,138,139,142]. In addition, much attention has also been paid to non-dairy products fermented with kefir grains. Due to the wide variety of sugars and subtracts used in the fermentation process, the microbial diversity of non-dairy kefir grains may be greater than that of traditional kefir grains. Several authors reported 45 different bacterial species and 23 yeasts [143]. However, due to its great complexity, the relative composition of bacteria and yeasts may vary during the kefir fermentation process, making it difficult to control and obtain uniform products for industrial use [143].

### 5.4. Application of EPS-Producing LAB in Plant-Based Beverages

In recent years, interest in vegetarian and vegan diets has increased for many reasons (e.g., health concerns, ethical concerns, sustainability). The challenge for manufacturers of plant-based milk alternatives is to produce products with acceptable taste and texture for customers [92,144]. The application of EPS-producing LAB has emerged to improve the sensory and organoleptic analysis of such products, as EPS can positively influence texture, mouthfeel, and syneresis [92,145]. Several studies have focused on the use of in situ production of EPS in fermented plant-based beverages, as it is possible to develop a large number of products with improved sensory properties. Li et al. [14] studied the fermentation process of soy milk with an EPS-producing *L. plantarum* strain over a period of 21 days. The fermented soy milk maintained the apparent viscosity and EPS content, exhibited satisfactory technological properties, and improved the taste of soy milk [14]. In another study, Hickisch et al. [144] attempted to produce a vegetable yoghourt alternative from lupins, which proved to be a good plant choice. A different strain of EPS-producing *Lactiplantibacillus plantarum* (TMW 1.460) was used for the fermentation process, which achieved better sensory values, such as appearance, texture, aroma, and taste [144]. Beverages mimicking the characteristics of cow’s milk yoghourt were also developed using an aqueous extract of quinoa flour and *Weissella* spp. as EPS producers and showed high acceptability in terms of acidity, sweetness, texture, and overall appearance [146,147].

### 5.5. Application of EPS in Bakery

EPS produced by LAB has been proposed as a promising alternative to replace the use of polysaccharides in bakeries as an additive for sourdough because they have thickening properties. EPS-producing LAB can be incorporated into fermented sourdough to have a positive effect on the techno-functional properties of baked goods [148,149,150]. The in situ production of EPS has been shown to improve the handling and stability of the dough, but also to increase the technological properties of the final product, such as the texture and volume of the bread [150,151]. Moreover, the use of selected EPS-producing LAB strains in sourdoughs has been investigated as a novel technological approach to compensate for quality losses in the functional properties of reduced-sugar products or to replace the added fat in bakery products [149,152]. In addition, some studies have shown that the use of EPS-producing LAB has a cold-protective effect and overcomes the quality loss of frozen dough products [153]. The use of polysaccharides has gained interest in recent years with the increasing production of gluten-free products. Not only are customers looking for healthier products, but people with a chronic autoimmune disease—celiac disease—are also looking to bakeries for better-quality gluten-free products [8,92,154]. The beneficial effect of EPS produced by LAB is to improve the structure and volume of gluten-free or gluten-containing bread, as they are able to bind water and form a high-quality network with other dough components. This later leads to softer bread crumb and longer shelf life [10,155]. The in situ production of EPS was demonstrated in a recent study by Zheng et al. [45], in which the EPS-producing *Limosilactobacillus reuteri* was used during the fermentation process. Its ability to produce fructans and glucans resulted in better-quality gluten-free bread.

### 5.6. Application of EPS in Meat Industry

While EPS-producing LAB are widely used in the dairy industry, their use in the meat industry is still a relatively new area of research [74]. Meat production dates back many millennia, and the use of lactic acid bacteria has given us an enormous variety of traditional foods around the world. By producing lactic acid or acetic acid in raw meat, we can control food safety, texture, color of meat, and much more [92,94]. Meat products contain many important nutrients (protein, iron, zinc, vitamins, etc.), but consumption of high-fat meat products may correlate with diseases such as diabetes or cardiovascular disease. Customer demand for low-fat meat products challenges the meat industry to develop new products to serve this market. This poses many problems for the meat industry because low-fat processed meat products may be deficient in terms of technological and sensory properties [74,92,94,156]. Hydrocolloids are widely used as food additives in the food industry. In the meat industry, they are known to improve the water-holding capacity and gelling properties of meat proteins and can improve the texture of low-fat meat products, but nowadays customers reject food additives on the ingredient list [157,158,159,160]. Therefore, in situ production of EPS by LAB during meat processing could be an interesting alternative. Few studies have been conducted on the in situ production of EPS by LAB in meat products. Meat products evaluated for the use of LAB for in situ production of EPS included cooked ham, raw fermented sausages (sucuk and salami), and spreadable raw fermented sausages [74]. Although the processing conditions for ham production showed a negative effect on EPS production, the use of in situ EPS-forming LAB was more promising for the production of fermented sausages [74,161]. In the study by Hilbig et al. [46], selected LAB strains (*L. plantarum* and *L. curvatus* strains) were able to produce sufficient amounts of EPS during sausage fermentation to allow reduction of the high fat content of fermented raw sausages [46]. Moreover, Dertli et al. [162] used EPS-forming LAB cultures in a Turkish fermented sausage to improve its textural properties, which became harder, less sticky, and tougher.

## 6. Health-Promoting Effects

Several pieces of evidence have shown that EPS produced by LAB are associated with numerous functional roles and health benefits, such as immunomodulatory, antioxidant, anticancer, antiulcer, anti-biofilm, and cholesterol-binding effects [5,20,163,164,165,166,167,168,169]. However, it has been found that the biological activity of different EPS is influenced by their chemical structure (main chain, branching and molecular weight) [170,171]. Fermented foods can provide LAB-derived EPSs with prebiotic properties that promote colonization of the gut with beneficial bacteria [90,172,173]. One of the beneficial capabilities of probiotic bacteria is the formation of biofilms that support their colonization and maintenance of their population in the harsh conditions of the human gastrointestinal tract (GIT) [174]. Thus, attachment of probiotic LAB to epithelial cells in the GIT has been shown to prevent colonization by pathogenic organisms, stimulate the host immune system, and protect epithelial cells from toxic compounds (e.g., carcinogens and toxic metal ions) [102,175]. In addition, LAB-derived EPS can reduce or inhibit biofilm formation by pathogenic bacteria, thereby preventing infectious diseases [175,176]. Other studies have shown that these EPS have the potential to affect the gastrointestinal tract by protecting intestinal cells from toxins and lowering cholesterol levels through increased secretion of bile acids [165,168,177].

**Figure 3:**
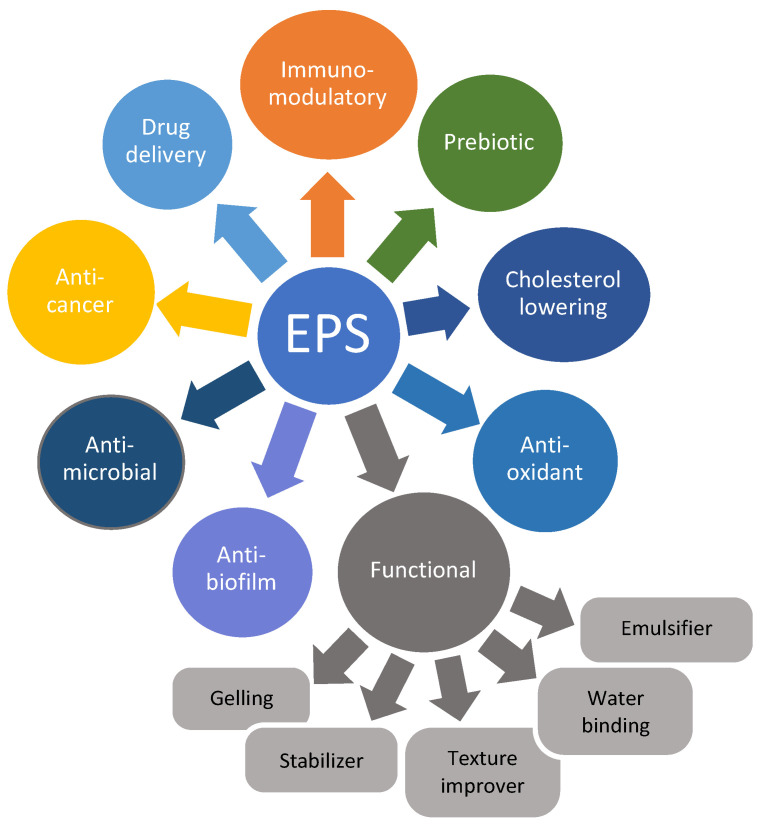
Biological and functional activities exerted by EPS produced by LAB.

The multiple functional activities of LAB-produced EPSs are also very useful and find application in therapeutic medicine as drug carriers and in adjuvant therapy for the treatment of inflammation and cancer due to their high ability to form hydrogels [92,178,179,180,181,182,183]. For example, Moscovici [184] attempted to incorporate biomolecules and therapeutics into the internal structure of EPS for use as an intelligent drug delivery system for the treatment of brain tumors or other neurological diseases due to their biocompatibility and apparent lack of toxicity. Most studies on the health benefits of EPS have been conducted in vitro, and there is limited information on in vivo experiments with oral administration [167]. However, as new EPS and EPS-producing LAB have been identified, there are emerging studies that provide positive evidence for the use of EPS in medicine and functional foods (Figure 3).

### 6.1. Prebiotic Activity

Although a growing number of studies confirm the health-promoting effects of EPS, no mechanism of action has yet been proposed that clearly explains how they can exert such activities on biological tissues [168,185]. However, some authors propose that EPSs remain longer in the gastrointestinal tract to exert their effects as prebiotics. Since EPSs are not degraded by human digestive enzymes, they can exert their prebiotic effect and promote colonization by probiotic bacteria, such as lactobacilli and bifidobacteria [20,169,186]. For example, Abedfar et al. [187] found that symptoms of lactose intolerance decreased with the presence of EPS in the gastrointestinal tract, which was attributed to the proliferation of lactase-producing lactobacilli. Hamdy et al. [188] evaluated the prebiotic activity of EPS in a rat model over 60 days and found an increase in *Lactobacillus* counts while *Escherichia coli* counts decreased, confirming that the increase in beneficial bacteria was related to the presence of EPS. Recently, Pan et al. [189] reported the modulation of the gut microbiota of mice by feeding a linear dextran produced by a strain of *Leuconostoc pseudomesenteroides*. EPS altered the structure of the gut microbiota and decreased the ratio of *Bacteroidetes* to *Firmicutes* in treated mice.

### 6.2. Immunomodulatory Activity

Many food components (called immunomodulators) are known to modulate the innate and adaptive immune system. EPSs, proteins, peptides, glycoproteins, lipopolysaccharides and others are among the many molecules that have immunomodulatory properties. These immunomodulators alter the activity of immune function by regulating cytokines and are able to suppress infections and prevent immunodeficiency-related diseases (e.g., inflammatory bowel disease) of the gastrointestinal tract [168,171,190,191,192]. Acidic HePS, characterized by having phosphate (i.e., a negative charge) in their position, seem to be well-able to trigger the immune response [193]. This has been confirmed by studies showing that phosphate is the molecule that triggers the immune response, as chemical dephosphorylation of these HePS leads to a decrease in the stimulatory effect [168,171,190,194,195]. However, immunomodulatory activity has also been associated with high molecular weight HePS, which appears to suppress the immune response [168,171,185,190,196,197,198]. In addition, the monosaccharide composition of EPS has been shown to influence its anti-inflammatory properties, possibly by association with the recognition of receptors on the surface of immune cells [199,200]. Several studies have shown that EPS produced by LAB stimulates the production of cytokines and antigen-presenting cells [201,202]. Dilna et al. [203] reported the promoting effect of EPS produced by *L. plantarum* on the production of cytokines by macrophages, especially tumor necrosis factor α (TNF-α), interleukin 6 (IL -6), IL -1b and IL -12. In a particular study, (1→6)-α-D-glucan with low Mw from *Lactobacillus confusus* was shown to modulate the immune system to produce the pro-inflammatory mediator nitric oxide (NO) and cytokines [195]. In a rat-induced allergic asthma model using ovalbumin (OVA), Domingos-Lopes et al. [204] demonstrated that the orally administered EPS producer *Leuconostoc citreum* induced immune tolerance to the allergen OVA, specifically by downregulating the overproduction of IgE and reducing the Th2-mediated allergic response. Moreover, in a study using a similar mouse model of respiratory allergy, intranasal administration of EPS from *Bifidobacterium longum* was shown to reduce the recruitment of eosinophils in the lungs [205]. In a similar study, EPS from *L. rhamnosus* was also shown to effectively control T-cell-dependent immune responses in various inflammatory diseases [206].

Other studies also demonstrated the important role of EPS in maintaining the balance of the immune system during infection or inflammation [185]. In the study by Matsuzaki et al. [207], it was shown that the EPS-producing *Leuconostoc mesenteroides* strain could induce the secretion of immunoglobulin A (IgA), indicating a potential use against mucosal pathogens.

### 6.3. Antioxidant Activity

The accumulation of reactive oxygen species (ROS) leads to oxidative stress in living organisms, which damages biological macromolecules such as DNA, RNA, proteins, and lipids, and can result in tissue damage that can later lead to the development or progression of various diseases (obesity, cancer, neurodegenerative disorders, etc.) [168,171,196,203,208,209]. Exogenous regulation with natural antioxidants (ascorbic acid, α-tocopherol and carotenoids) has been proposed as a way to reduce oxidative stress and delay oxidative damage. Therefore, EPS derived from LAB can be considered as effective natural antioxidants for preventing oxidative stress caused by free radicals. There are numerous studies reporting the antioxidant activity of EPSs produced by LAB, which were evaluated based on their ability to scavenge superoxide anions and hydroxyl radicals [210]. A recent study by Liu et al. [211] reported that sulfonation of EPS produced by *Lactiplantibacillus plantarum* increased antioxidant activity, thereby protecting epithelial cells.

### 6.4. Cholesterol Lowering Abilities

Major risk factors for cardiovascular disease include high blood pressure and elevated blood cholesterol. There are a growing number of studies showing the ability of EPS from LAB to regulate serum cholesterol levels through intestinal adsorption of this molecule [185]. In this sense, Soh et al. [212] showed the adsorption of cholesterol through an in vitro enzymatic reaction and a polysaccharide precipitation process. Studies in animals and humans have also shown the effect of LAB-derived EPS in lowering blood cholesterol levels. For example, Dilna et al. [203] showed that EPS isolated from *L. plantarum* RJF4 had a cholesterol-lowering effect. In another study, mucilage EPS produced by *L. lactis* subsp. *cremoris* was shown to have a beneficial effect on cholesterol metabolism in rats by lowering serum cholesterol levels [213]. In a similar study, Ai et al. [214] showed that EPS has an antihypertensive effect in an in vivo rat model.

### 6.5. Anti-Biofilm Formation

Many bacterial species, including pathogenic bacteria, become more resistant to extracellular stress conditions by building biofilms [174]. Biofilms are arrays of bacterial cells bound to a surface in an extracellular polymer matrix composed of EPS, proteins, and extracellular DNA [176]. Biofilms formed by pathogenic bacteria can cause many problems, such as food safety issues or ineffective treatment of infectious diseases [168,215,216].

Several authors reported the potential of LAB -derived EPS to reduce or prevent the formation of biofilms by pathogenic bacteria and thereby control infections caused by the pathogens [176]. For example, an HePS composed of mannose, glucose and galactose was shown to have considerable anti-biofilm activity against six bacterial pathogens [217]. Several authors have proposed different mechanisms by which EPS can inhibit biofilm formation. Lynch et al. [10] hypothesized that EPS may mask adhesion molecules in the intestinal mucus, thus preventing adhesion of other bacteria. On the other hand, Abdalla et al. [176] proposed that biofilms produced by LAB promote their own colonization in the host mucosa, thus inhibiting the formation of biofilms by bacterial pathogens.

### 6.6. Antimicrobial Activity

In recent years, antimicrobial resistance has become a very serious health problem, increasing the demand for new antimicrobial drugs. Several authors have shown that LAB -derived EPSs can exhibit antagonistic effects in vitro against Gramme-positive and Gramme-negative pathogens or against pathogenic bacteria in the gastrointestinal tract [47,176,218]. For example, HePS produced by *Lactobacillus gasseri* inhibited in vitro antibacterial activity against several foodborne pathogens such as *Escherichia coli*, *Listeria monocytogenes* and *Staphylococcus aureus* [76]. The EPS produced by a *Lactobacillus* strain also showed considerable antibacterial activity against the pathogenic bacteria *Salmonella enterica* and *Micrococcus luteus* [219]. In a similar study, HePS isolated from *L. kefiranofaciens* was shown to exert bactericidal activity against *Listeria monocytogenes* and *Salmonella enteritidis* [220]. In another study, silver nanoparticles with EPS produced by LAB were used against pathogenic bacteria. These were found to have antimicrobial activity against Gramme-positive and Gramme-negative bacteria, with Gramme-positive bacteria being more sensitive [221]. The antimicrobial activity of EPSs in vivo can also be explained by the prebiotic effect that helps probiotics to colonise the surface of the gastrointestinal tract, thus enhancing their competitive inhibition of pathogenic bacteria in the host [90]. Recent studies showed remarkable antifungal activity of some EPS produced by *Lactobacillus* strains [176]. Moreover, Álvarez et al. [222] used the strain *L. plantarum* for the production of EPSs included in an edible coating as an antifungal agent. EPSs have also been reported to exert antiviral activities, especially the sulfated EPSs [90]. It is believed that the immunomodulatory effect of EPSs produced by LAB is responsible for the antiviral activity [176,182]. In a study by Nagai et al. [223], EPS produced by *Lactobacillus bulgaricus* in fermented yoghourt was fed to mice infected with influenza virus. They found an increase in natural killer cell activity and an increase in immunoglobulins IgG1 and IgA, which led to a reduction in viral infection.

### 6.7. Anti-Cancer Activity

Cancer is an abnormal form of tissue growth due to irreversible damage to DNA caused by mutations in certain proto-oncogenes and tumor suppressor genes, resulting in a progressive increase in the number of dividing cells. Highly effective chemotherapies are cytotoxic/immunotoxic, which affects tumor development and impairs patient recovery [168,224]. The discovery and identification of new antitumor drugs with low side effects on the immune system has become an important goal in many immunopharmacology studies. EPS from safe natural sources such as LAB usually have low cytotoxicity and side effects and can serve as good substitutes for synthetic antitumor agents [196,216,224]. Liu et al. [202] demonstrated the antiproliferative effect of *L. casei* EPS on HT -29 cell line. Al-so, the EPS produced by *L. plantarum* strain showed remarkable antitumor activity on HepG-2, BGC-823 and especially HT -29 cancer cells [216]. EPS produced by *L. casei*, *L. plantarum* and *L. acidophilus* showed antitumor properties against different cell lines depending on the dose [225]. Self-assembled nanoparticles from EPS loaded with anticancer drugs, such as epirubicin, increase the uptake of the drug in mice bearing tumors while decreasing the uptake in heart and kidney. They show a sustained release pattern and a 70% reduction in tumor volume [226]. Another study by Liu et al. [208] showed the antioxidant and antiproliferative effects of EPS produced by LAB on hepatoma cells, HepG2.

### 6.8. Drug Delivery Systems

EPSs are promising candidates for drug delivery systems due to their bioactive function and ability to transport drugs. Bacterial EPSs not only serve as bioactive agents but can also be potential carriers for valuable drugs, including growth factors and antitumor agents. Although their function as carriers is similar, the production of EPSs as drug carriers is simpler than the production of biological scaffolds loaded with viable cells. As drug carriers, EPS can be modified to facilitate the controlled release of drugs, increase the shelf life of drugs in the body, and improve drug efficacy. Antibiotics are often used as a model for drug release by bacterial EPS [168,227,228]. In a study by Wang et al. [229], it was reported that encapsulation with kefiran was able to protect ciprofloxacin from gastric conditions. In another study, sulfated EPS of bacterial origin were used as vaccine adjuvants for the treatment and prevention of hepatitis B virus in animal models [230].

## 7. Conclusions

The diversity of EPS produced by LAB has increased and become the focus of many researchers in recent years. The non-toxic behavior of EPSs offers tremendous opportunities for applications in food, medicine, and even pharmaceuticals. LAB -derived EPSs hold remarkable properties, improving stability, flavor, texture, shelf life, and rheological properties of several food products. In addition to technological benefits, recent research shows the health benefits of EPSs, such as immunomodulatory, antioxidant, prebiotic, antibiofilm, cholesterol-lowering, antimicrobial, and anticancer activities. Therefore, the use of LAB cultures for in situ production of EPS meets the consumer demand for health-promoting functional foods. The major limitation for the industrial use of EPS is the low yield and wide variation in the production capacity of EPS-producing strains. Therefore, it is crucial to explore new ideas to optimize the production of EPS for its future industrial use. The type of EPS (HoEPS or HeEPS), molecular weight, monosaccharide composition, and functional groups are also crucial factors affecting technological and biological activities. Most of the research conducted in recent years has focused on the evaluation of EPS structures as well as novel functionalities. However, the mechanisms by which EPSs exert their effects in biofilm formation, immune system modulation, and pathogen inhibition are very complex and still not fully understood. A better understanding of the underlying mechanisms could lead to the development of tailored EPSs with desired/specific properties for use in functional foods or as therapeutic agents.

## Figures and Tables

**Table 1:** Homopolysaccharides produced by lactic acid bacteria.

HoPS		LAB	Mw/Structure	Reference
α-D-glucans	Dextran	*Leuconostoc mesenteroides* *Leuconostoc citreum* *Leuconostoc pseudomesenteroides* *Lentilactobacillus parabuchneri* (formerly *Lactobacillus parabuchneri*) *Limosilactobacillus fermentum* (formerly *Lactobacillus fermentum*) *Limosilactobacillus reuteri* (formerly *Lactobacillus reuteri*) *Latilactobacillus sakei* (formerly *Lactobacillus sakei*) *Latilactobacillus curvatus* (formerly *Lactobacillus curvatus*) *Lactobacillus hordei* *Lactobacillus nagelli* *Lactobacillus mali* *Lactobacillus satsumensis* *Weissella confusa* *Weissella cibaria* *Streptococcus mutans* *Streptococcus salivarius*	Mw: 10^3^–10^7^ Da α-D-Glc(1,6)	[29,30,31,32,33,34,35,36,37,38,39,40,41,42,43,44,45,46,47]
	Mutan	*Limosilactobacillus reuteri* *Streptococcus downei* *Streptococcus mutans* *Streptococcus salivarius*	Mw: >10^6^ Da α-D-Glc(1,3)	[35,36,40,48,49,50]
	Alternan	*Leuconostoc mesenteroides* *Streptococcus salivarius* *Leuconostoc citreum*	Mw: >10^6^ Da (α-D-Glc(1,6)/α-D-Glc(1,3)	[51,52,53]
	Reuteran	*Limosilactobacillus reuteri*	Mw: 10^7^ Da α-D-Glc(1,4)/α-D-Glc(1,6)	[40,54]
β-Glucans		*Lactobacillus suebicus* *Pediococcus parvulus*	Mw: 10^5^–10^6^ Da [β-D-Glc(1,3) with side chain linked (1,2)]	[55,56]
Fructans	Levans	*Leuconostoc mesenteroides* *Limosilactobacillus reuteri* *Streptococcus mutans* *Bacillus subtilis*	Mw: 10^4^–10^8^ Da β-D-Fru(2,6)	[47,57]
	Inulin-type	*Streptococcus mutans* *Limosilactobacillus reuteri* *Leuconostoc citreum* *Lactobacillus johnsonii*	Mw: 10^3^–10^7^ Da β-D-Fru(2,1)	[20,55,58,59]
Polygalactan		*Lactococcus lactis* *Lactobacillus delbruecki*	pentameric repeating unit of galactose	[47]

**Table 2:** Heteropolysaccharides produced by lactic acid bacteria.

LAB	HePS Composition	Molecular Weight	Reference
*Streptococcus thermophilus* CC30	Glucose, galactose	58 to 180 kDa	[67]
*Streptococcus thermophilus* CH101	Glucose, galactose	8.5 × 10^5^ Da	[68]
*Streptococcus thermophilus* LY03	Glucose, galactose, N-acetylgalactosamine	1.8 × 10^6^ Da	[68]
*Streptococcus thermophilus* S-3	N-acetylgalactosamine, galactose, glucose	5.7 × 10^5^ Da	[69]
*Streptococcus thermophilus* NIZO2104	Galactose, ribose, N-acetylgalactosamine, glucose	0.9 × 10^6^ Da	[70,71]
*Streptococcus thermophilus* AR333	Galactose, glucose, galactosamine	3.1 × 10^5^ Da	[72]
*Lactobacillus delbruecki* subsp. *bulgaricus* CNRZ 1187	Rhamnose, arabinose, mannose, galactose, glucose	10^4^–10^6^ Da	[73,74]
*Lactobacillus delbruecki* subsp. *bulgaricus* DGCC291	Glucose, galactose	1.4 × 10^6^ Da	[70,71]
*Lactobacillus delbruecki* subsp. *bulgaricus* NCIMB702074	Glucose, galactose	1.8 × 10^6^ Da	[70,71]
*Lacticaseibacillus casei* (formerly *Lactobacillus casei*) WXD030	Glucose, glucosamine, mannose	37.37 kDa	[75]
*Lactobacillus gasseri* FR4	Glucose, mannose, galactose, rhamnose, fucose	1.9 × 10^5^ Da	[76]
*Lactobacillus helveticus* MB2-1	Glucose, mannose, galactose, rhamnose, arabinose	1.83 × 10^5^ Da	[77]
*Lactobacillus kefiranofaciens* WT-2B	Kefiran: glucose, galactose	7.6 × 10^5^ Da	[78]
*Lactobacillus johnsonii* 142	D-glucose and D-dalactose	1.0 × 10^5^ Da	[79]
*Latilactobacillus sakei* O-1 (formerly *Lactobacillus sakei*)	Glucose, rhamnose	6 × 10^6^ Da	[80]
*Lactiplantibacillus plantarum* (formerly *Lactobacillus plantarum)* JLK0142	Glucose, galactose	1.34 × 10^5^ Da	[81]
*Lactiplantibacillus plantarum* WLPL04	Xylose, glucose, galactose	6.61 × 10^4^ Da	[82]
*Lactiplantibacillus plantarum* YW11	Glucose, galactose	1.1 × 10^5^ Da	[83]
*Lactiplantibacillus plantarum* JLAU103	Arabinose, rhamnose, fucose, xylose, mannose, fructose, galactose, glucose	12.4 kDa	[84]
*Lactiplantibacillus plantarum* EP56	Glucose, galactose, rhamnose	8.5×10^5^ Da	[85]
*Lactiplantibacillus plantarum* C88	Glucose, galactose	1.2 × 10^6^ Da	[86]
*Lactiplantibacillus plantarum* C70	Arabinose, mannose, glucose, galactose	3.8 × 10^5^ Da	[87]

## Data Availability

Not applicable.
